# Role of Eryptosis in Hemorrhagic Stroke

**DOI:** 10.3389/fnmol.2022.932931

**Published:** 2022-07-28

**Authors:** Mei Fang, Fan Xia, Yuqi Chen, Yuke Shen, Lu Ma, Chao You, Chuanyuan Tao, Xin Hu

**Affiliations:** ^1^Department of Neurosurgery, West China Hospital, Sichuan University, Chengdu, China; ^2^West China School of Public Health, Sichuan University, Chengdu, China

**Keywords:** subarachnoid hemorrhage, erythrocytes, intracerebral hemorrhage, hemorrhagic stroke, eryptosis

## Abstract

Erythrocytes undergo certain morphological changes resembling apoptosis during senescence or in an abnormal state/site, which is termed eryptosis. This process is characterized by phosphatidylserine (PS) exposure, membrane blebbing, and cell shrinkage. Eryptotic erythrocytes are subsequently removed *via* macrophage-mediated efferocytosis. In hemorrhagic stroke (HS), blood within an artery rapidly bleeds into the brain tissue or the subarachnoid space, resulting in severe neurological deficits. A hypoxic, over-oxidative, and pro-inflammatory microenvironment in the hematoma leads to oxidative stress, hyperosmotic shock, energy depletion, and Cl^–^ removal in erythrocytes, which eventually triggers eryptosis. In addition, eryptosis following intracerebral hemorrhage favors hematoma clearance, which sheds light on a common mechanism of intrinsic phagocytosis. In this review, we summarized the canonical mechanisms of eryptosis and discussed its pathological conditions associated with HS. Understanding the role of eryptosis in HS may uncover additional potential interventions for further translational clinical research.

## Introduction

It has been suggested that the lifespan of mature erythrocytes varies between 100 and 120 days *in vivo* (Lang et al., 2012). Senescent erythrocytes are removed from the circulation by macrophages present in the spleen, the liver, and the bone marrow (Lang et al., 2012). However, it has been confirmed that erythrocytes, similar to nucleated cells, undergo programmed cell death, referred to as eryptosis (Lang et al., 2012). Although enucleated erythrocytes cannot exhibit mitochondrial depolarization or nuclear condensation (hallmarks of apoptosis in nucleated cells), they still exhibit the typical features of apoptosis, such as phosphatidylserine (PS) exposure, cell shrinkage, and membrane blebbing (Berg et al., 2001; Bratosin et al., 2001; Elmore, 2007). Eryptotic erythrocytes are rapidly recognized by circulating macrophages with specific phagocyte-associated receptors, and then, macrophage-mediated efferocytosis occurs and the engulfed erythrocytes are subsequently degraded and recycled (Larsson et al., 2016; Tao et al., 2020; Wei et al., 2020).

The sudden onset of neurological deficits caused by the rupture of cerebral vessels and subsequent bleeding into or around the brain tissue is defined as hemorrhagic stroke (HS). The pathophysiology of brain injury after HS is chiefly due to intracerebral hematoma mass effect or subarachnoid space clots, as well as the subsequent secondary brain injury induced by peripheral and clot-derived factors (Hemphill et al., 2015; Bian et al., 2020). Erythrocytes that are not phagocytosed by microglia or macrophages may lyse and release potentially harmful components (e.g., hemoglobin/iron, CA1, and Prx2) into the extracellular space, damaging neurons, endothelial cells, pericytes, and astrocytes, which are the main components of the neurovascular units (NVU), thus leading to NVU dysfunction (Lee et al., 2010; Bian et al., 2020; Sun et al., 2021; Holste et al., 2022). Therefore, enhancing the endogenous clearance of “intact” erythrocytes within clots through the innate phagocytic system or limiting hemolysis are therapeutic approaches that can effectively attenuate secondary brain injury after HS (Wilkinson et al., 2018; Holste et al., 2021; Wang et al., 2021). Abnormal erythrocytes, such as those within hematoma/subarachnoid clots, are exposed to additional stress that may trigger eryptosis in some of these erythrocytes (Chang et al., 2018). Furthermore, since efferocytosis of eryptotic erythrocytes is also a way of “intact” removal of erythrocytes, enhanced eryptosis within the bleeding site may attenuate neurological deficit after HS (Chang et al., 2018; Liddle et al., 2020). In this review, we compiled the present knowledge on the cellular mechanisms of eryptosis and specifically focused on the relationship between eryptosis and HS.

## Eryptosis

### Cellular Mechanisms

**FIGURE 1:**
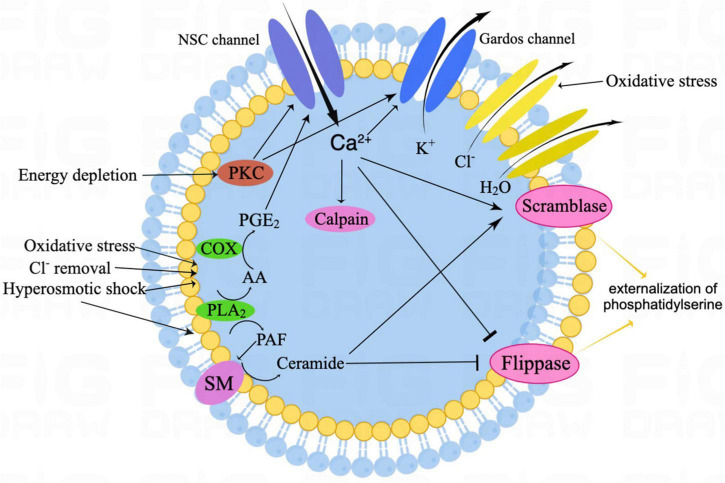
The schematic illustration of canonical eryptosis signaling. Eryptosis is mainly stimulated by increased intracellular Ca^2+^ and ceramide. Among oxidative stress, with Cl^–^ removal and hyperosmotic shock, activated PGE_2_ stimulates NSC channels, thus resulting in Ca^2+^ influx. In addition, hyperosmotic shock also leads to ceramide formation. Then, increased cytosolic Ca^2+^ activates Gardos channels, resulting in K^+^ efflux, together with Cl^–^ efflux and osmotically H_2_O outflow, which eventually leads to cell shrinkage. Increased cytosolic Ca^2+^ also leads to the translocation of phosphatidylserine (PS) from the inner leaflet of the cell membrane to the erythrocyte surface by activating the scramblase and inhibiting the flippase. In addition, increased cytosolic Ca^2+^ further mediates the activation of the calpain, which leads to cell membrane blebbing. Energy depletion triggers eryptosis, mainly by PKC-dependent membrane proteins phosphorylation. NSC, non-selective cation; SM, sphingomyelinase; PKC, protein kinase C; COX, cyclooxygenase; PGE_2_, prostaglandin E2; AA, arachidonic acid; PLA_2_, phospholipase A2; and PAF, platelet-activating factor.

Figure 1 shows the signaling pathways involved in eryptosis. Eryptosis is mainly initiated by increased cytosolic Ca^2+^ concentrations when triggered by some xenobiotics and endogenous stimulators (Bratosin et al., 2001). Ca^2+^ enters erythrocytes through Ca^2+^ permeable non-selective cation channels on the erythrocyte membrane (Lang et al., 2012), and the TRPC6 (transient receptor potential channel 6) is considered to be partly involved (Foller et al., 2008). Cation channels are stimulated by prostaglandin E_2_ (PGE_2_) generated from membrane phospholipids due to hyperosmotic shock, oxidative stress, and extracellular Cl^–^ removal (Lang et al., 2005b). The increased cytosolic Ca^2+^ activates Ca^2+^ -sensitive K^+^ channels called Gardos channels, which lead to cell shrinkage due to the loss of KCl with osmotically outflowing water (Lang and Lang, 2015; Pretorius et al., 2016). Thus, osmotic cell shrinkage activates cation channels and accelerates erythrocyte Ca^2+^ uptake (Lang et al., 2006). Elevation of intracellular Ca^2+^ also leads to the translocation of PS from the inner layer of the cell membrane to the erythrocyte surface by activating scramblase and inhibiting flippase (Weiss et al., 2012). The efflux of K^+^ through the Gardos channel enhances PS exposure. The externalized PS on the cell surface may encourage phagocytosis by macrophages and allow clearance of erythrocytes before the onset of hemolysis. Increased cytosolic Ca^2+^ further mediates the activation of calpain, a cysteine endopeptidase that degrades the membrane proteins [e.g., anion exchanger1 (AE1)], and induces cell membrane blebbing (Pant et al., 1983). Oxidative stress is a major cause of eryptosis; in addition to triggering eryptosis by activating Ca^2+^- permeable cation channels, it activates Cl^–^ channels on the erythrocyte membrane, which are necessary for subsequent erythrocyte shrinkage (Huber et al., 2002; Bissinger et al., 2019). Moreover, caspases are also activated during oxidative stress, and they cleave the AE1 and stimulate PS exposure on the erythrocyte surface, although they are not necessary to induce eryptosis (Mandal et al., 2002, 2003).

Eryptosis can be stimulated by ceramide (Lang et al., 2010). During osmotic cell shrinkage, platelet-activating factor (PAF) is released from the erythrocytes membrane by activated phospholipases (Lang et al., 2005c). Erythrocytes express the PAF receptor, therefore, increased PAF further stimulates sphingomyelinase within erythrocytes, which can in turn lead to sphingomyelin breakdown, ceramide formation, and subsequent eryptosis (Lang et al., 2005c). Ceramide enhances the eryptotic effect by increasing the sensitivity of erythrocytes to cytosolic Ca^2+^ concentration rather than the change in Ca^2+^ concentration within the cytoplasm (Lang et al., 2004a).

Energy depletion is another crucial trigger for eryptosis (Klarl et al., 2006). Energy depletion-induced eryptosis is mainly mediated by the activation of protein kinase C (PKC) and the subsequent PKC-dependent membrane protein phosphorylation, which allows Ca^2+^ influx and K^+^ efflux, leading to Ca^2+^-dependent downstream pathways and osmotic shrinkage, respectively (Klarl et al., 2006; Lang et al., 2008). Moreover, energy depletion further stimulates eryptosis through the activation of Janus-activated kinase 3 (JAK3) and casein kinase 1α (CK1α activated in response to oxidative stress). The energy depletion may also compromise GSH synthesis, thus interfering with anti-oxidative defense (Lu, 2013). In addition to the abovementioned inducers, eryptosis could also be triggered by ligation of some specific receptors (e.g., the thrombospondin-1 receptor CD47 and the death receptor CD95/Fas) and several kinases (e.g., p38 mitogen-activated protein kinase) (Head et al., 2005; Mandal et al., 2005; Lang et al., 2015).

There are known inhibitors of eryptosis. Erythropoietin (EPO) counteracts the cation channels and Ca^2+^ entry and subsequently stimulates eryptosis (Myssina et al., 2003). Accordingly, the addition of extrinsic EPO exerted protective effects against oxidative stress-induced eryptosis in erythrocytes (Vota et al., 2012; Sun et al., 2018). However, enhanced eryptosis was found in transgenic animals overexpressing EPO, which may be explained by the fact that EPO could further activate genes in erythrocyte precursors, which renders erythrocytes more sensitive to eryptosis (Föller et al., 2007). NO is another potent inhibitor of eryptosis. It is partially effective and affects the downstream Ca^2+^, and it also stimulates cGMP-dependent protein kinase type I (cGKI), an eryptosis inhibitor (Lang et al., 2015). Nevertheless, higher concentrations of NO donors (e.g., nitroprusside) stimulate eryptosis, probably because of NO-induced oxidative stress (Lang and Lang, 2015). In addition, eryptosis can be inhibited by endothelin 1, endothelin B receptor agonist, catecholamines, adenosine, and several kinases (e.g., energy-sensing AMP-activated kinase and p21-activated kinase 2) (Lang et al., 2005a,^2012^, ^2015^; Niemoeller et al., 2007; Föller et al., 2010).

### Clinical Significance

As mentioned above, eryptosis tend to achieve “intact” removal of defective erythrocytes from circulating blood, which would otherwise die from hemolysis. In malaria, plasmodium parasitized within erythrocytes may cause oxidative stress in the host, stimulate oxidant-sensitive non-selective Ca^2+^-permeable cation channels, and trigger eryptosis (Föller et al., 2009). Thus, enhanced eryptosis is a protective mechanism against malaria that can achieve pathogen pro-clearance through phagocytosis of the host cells.

However, enhanced eryptosis can have unfavorable effects under certain conditions. The most common pathology of eryptosis is anemia, which impedes adequate oxygen supply to tissues (Lang et al., 2012). PS exposure on the cell surface of eryptotic erythrocytes forces them to bind to respective receptors of phagocytic cells that engulf, degrade, and rapidly clear erythrocytes from the circulation. Accelerated loss of erythrocytes by eryptosis uncompensated by enhanced erythropoiesis can lead to anemia (Lang et al., 2008). Many clinical studies have shown that eryptosis contributes to disease and drug-related anemia to a certain extent (Lang et al., 2008, 2015, 2017). In addition to its significant role in the phagocytosis of erythrocytes, PS exposure further impels erythrocytes’ adhesion to the endothelial cells of the vascular wall, partly by a membrane-bound form of chemokine CXCL16/SR-PSO (Borst et al., 2012; Weisel and Litvinov, 2019). In a mouse model of renal ischemia, eryptotic erythrocytes were found adjacent to the vessel wall in the renal medulla, indicating cytoadherence to the endothelium (Lang et al., 2004b). Furthermore, eryptotic erythrocytes also adhere to platelets, and the exposed PS provides a matrix for the assembly of coagulation enzymes, promotes thrombin formation, and contributes to microvascular occlusion (Lang et al., 2015; Weisel and Litvinov, 2019). Therefore, excessive eryptosis may also impede microcirculation.

## Enhanced Eryptosis Risk Factors of Hemorrhagic Stroke

### Diabetes Mellitus

Anemia occurs in 14–45% of patients with diabetes mellitus (DM). There is increasing evidence that enhanced eryptosis may be involved and may partly contribute to this condition (Maellaro et al., 2013; Gauci et al., 2017; Restivo et al., 2021). Glycoxidation, a hallmark of DM, can lead to the excessive formation and accumulation of advanced glycation end-products (AGEs) in erythrocytes, forcing them to lose their standard discoid shape and become acanthocytes (Vlassara and Uribarri, 2014). Carboxymethyl-lysine (CML) and carboxyethyl-lysine (CEL) promote morphological modifications if eryptosis-related erythrocytes through their pro-oxidant properties (Vlassara and Uribarri, 2014). Another AGEs, methylglyoxal (MG), may induce eryptosis by impairing energy production and antioxidative defense (Nicolay et al., 2006). In addition to glycoxidation, upregulated intracellular ceramide and caspase-3 activation also contribute to DM-induced eryptosis (Maellaro et al., 2013; Kempe-Teufel et al., 2018).

### Hypertension

Some studies indicated that arterial hypertension (AH) is closely associated with oxidative stress; for example, erythrocytes from patients with AH exhibit elevated lipid peroxidation and higher levels of 8-isoprostanes in their plasma (Pinzón-Díaz et al., 2018). Moreover, compared with healthy subjects, untreated patients with AH showed low activity of a few antioxidative enzymes, such as catalase, superoxide dismutase (SOD), and glutathione peroxidase (GSH-Px), as well as a lower concentration of glutathione in erythrocytes and blood (Pinzón-Díaz et al., 2018; Restivo et al., 2021). Since anti-hypertensive drugs (captopril and enalapril) also have an antioxidant effect, they can limit oxidative damage. Compared to untreated patients with AH, treated patients showed lower lipid peroxidation in erythrocytes and lower oxidative damage in plasma (Donmez et al., 2002; Pinzón-Díaz et al., 2018). A recent clinical study found that erythrocytes from patients with AH showed higher lipid peroxidation, higher intracellular Ca^2+^ concentration, and PS externalization on the cell membrane than those from patients without AH, indicating the enhanced eryptosis in these patients (Pinzón-Díaz et al., 2018).

### Dyslipidemia and Atherosclerosis

Erythrocytes can be entrapped within atherosclerotic lesions at the site of intraplaque hemorrhage, where they can be engulfed by macrophages, and their modulatory role in the development of atherosclerosis has been clarified (Unruh et al., 2015). A recent clinical study demonstrated that patients with dyslipidemia showed higher PS externalization on the erythrocyte membrane than healthy individuals (Pinzón-Díaz et al., 2018). Moreover, erythrocytes from high-fat diet-fed mice showed an increased level of PS externalization as well as enhanced erythrophagocytosis *in vitro*, which may implicate the additional pro-atherogenic role of erythrocytes in obesity (Unruh et al., 2015). The mechanisms underlying enhanced eryptosis in patients with dyslipidemia are mainly associated with oxidative stress, as erythrocytes from such patients showed higher lipid peroxidation and lower concentrations of glutathione when compared to healthy subjects (Pinzón-Díaz et al., 2018). Thus, both oxysterols and 4-hydroxy-*trans*-2-non-enal (cholesterol and fatty acids oxidation by-products, respectively) have demonstrated eryptosis induction in healthy human erythrocytes at physiological concentrations, mainly through the elevation of PGE_2_ concentration within erythrocytes (Tesoriere et al., 2014; Allegra et al., 2020).

## Enhanced Eryptosis Within Hemorrhagic Stroke

During HS, the blood flow to the brain tissue is interrupted, so the involved brain tissue is deprived of oxygen, glucose, and other indispensable nutrients (Borgens and Liu-Snyder, 2012). The high metabolic nature of the brain makes it to be more vulnerable to hypoxia caused by decreased cerebral blood flow, increased intracranial pressure, and edema (Borgens and Liu-Snyder, 2012). In the central nervous system (CNS), primary injury results from mechanical damage, and its effect is usually limited and localized if the lesion is not further amplified by a secondary injury. Secondary injury (e.g., the neurotoxic effects of hemolysis products) usually leads to irreversible damage to the NVU, blood-brain-barrier disruption, and deadly brain edema. Hemolysis products cause oxidative stress after HS, which may elicit additional neuronal loss, brain edema, and NVU dysfunction (Xi et al., 2006). Similarly, intracerebral infusion of hemoglobin and its degradation products have also been shown to cause brain damage, and inhibition of heme oxygenase and administration of deferoxamine (an iron chelator) effectively attenuates ICH-induced brain injury (Huang et al., 2002; Koeppen et al., 2004; Zeng et al., 2018). To avoid the cytotoxicity of erythrocyte components, a possible approach is to accelerate the “intact” removal of erythrocytes (e.g., erythrophagocytosis) before hemolysis. However, only a few studies confirmed the occurrence of eryptosis in the brain following HS. In a study on the role of macrophages in the recovery after ICH, CD45^–^TER119^+^ erythrocytes were found to be the major cell types that presented PS externalization on the cell membrane in the perihematomal region, and the number of PS^+^ erythrocytes (eryptotic) was about 30-fold more than that in the leukocytes at day 3 after ICH (Chang et al., 2018). In acute ischemic stroke, the initial level of PS exposure on erythrocytes in the circulation was 2.40-fold higher than that in the control group, and PS positive erythrocytes continuously increased within the first week (Yao et al., 2017). In neurodegenerative diseases, such as Parkinson’s disease (PD), it was found that most erythrocytes from patients with PD presented a typical eryptotic shape (Pretorius et al., 2014). In addition, decreased erythrocyte membrane elasticity in patients with PD was detected compared to that in healthy individuals, suggesting phospholipid membrane scrambling of erythrocytes in these patients (Pretorius et al., 2014).

Interestingly, we found a considerable overlap of triggers between secondary brain injury and eryptosis, such as oxidative stress, energy depletion, and inflammation, suggesting that cellular mechanisms involved in secondary brain injury may also cross-act with erythrocyte homeostasis within the bleeding site. Oxidative stress plays a prominent role in the pathogenesis of HS and is a potent trigger for eryptosis (Lee et al., 2010; Aronowski and Zhao, 2011; Bissinger et al., 2019). The hemolysis of erythrocytes in extravasated blood is not synchronous. Conversely, the core of the hematoma is particularly vulnerable to hemolysis owing to interrupted blood flow and subsequent energy depletion (Xia et al., 2022). This means that the early production of heme and iron can produce oxidative stress in the surrounding non-hemolytic erythrocytes. In addition, disrupted mitochondrial respiration following periods of ischemia, such as those that follow SAH (e.g., global ischemia during early brain injury and vasospasm during delayed brain injury), is another leading contributor to oxidative stress by producing excessive free radicals (Ayer and Zhang, 2008). Additionally, endogenous antioxidant systems (both enzymatic and non-enzymatic), such as SOD and GSH-Px, are inhibited in HS; therefore, adequate elimination of generated excess free radicals does not occur (Ayer and Zhang, 2008; Chen et al., 2014). Hypermetabolism has been detected in the perihematomal region after ICH, and two possible explanations include stimulation of glycolysis by ischemia or neuronal and glial cell excitations by excitatory amino acids (Ardizzone et al., 2004). Anaerobic glycolysis is a less efficient means of producing ATP from glucose with the production of only 2 ATPs per glucose molecule, and glucose may be rapidly consumed in the perihematomal region (Ardizzone et al., 2004). Under conditions of glucose deficiency, the lack of glucose for the energy metabolism of erythrocytes can lead to energy depletion that can also induce eryptosis. In addition, regional ischemia and cortical spreading depolarization (CSD) during SAH can contribute to regional brain glucose depletion (Vergouwen et al., 2008; Feuerstein et al., 2010). It is well-known that the activation of the complement cascade and the subsequently formed terminal complex (C5b-9) play a crucial role in hemolysis after HS (Cao et al., 2016; Wang et al., 2019, 2021; Xia et al., 2022). However, the complement is also a trigger of eryptosis, and there is evidence that the sub-lytic terminal complement complexes C5b/6, C5b-7, and especially C5b-8, can lead to eryptosis by inducing ceramide generation and Ca^2+^ influx (Balola et al., 2019). Furthermore, the PS on the erythrocyte membrane decreased after C9 assembly and MAC formation, which may be due to hemolysis of eryptotic RBCs or inhibition of eryptosis by C9 (Balola et al., 2019). After HS, microglia are activated immediately as they respond to extravasated blood, and these inflammatory cells, in turn, produce pro-inflammatory chemokines and cytokines, which leads to the recruitment of peripheral immune cells (Tschoe et al., 2020). Furthermore, the erythrocyte membrane structure can also respond to inflammatory molecule insults (e.g., cytokines), especially IL-8, resulting in the pathological deformability typically seen in eryptosis (Bester and Pretorius, 2016).

Except within the hematoma or subarachnoid clot, eryptosis may also affect the lifespan of circulating erythrocytes of patients with HS. Eryptosis is augmented in several diseases (such as heart failure, chronic kidney disease, and sepsis), and researchers focused on its impact on disease-related anemia (Kempe et al., 2007; Lang et al., 2015). The incidence rates of anemia at admission in patients with ICH and SAH were approximately 5.5 and 25.8%, respectively, and anemia is associated with an increased mortality risk of stroke (Kumar et al., 2009; Li et al., 2016; English et al., 2018). Although there is no evidence of eryptosis of circulating erythrocytes in patients with HS, we suppose that HS-associated anemia may be compounded by eryptosis, as increased circulating PS-exposed erythrocytes were detected in patients with acute ischemic stroke (Yao et al., 2017). Further studies are required to confirm this hypothesis.

## Discussion

Enhancing the endogenous clearance of erythrocytes without lysis and the subsequent release of harmful substances are ideal alternative strategies for ICH treatment. Erythrocytes express CD47 on their surface as a “do not eat me” signal, which helps macrophages/microglia or dendritic cells (DCs) to discriminate “self” from “non-self” by using an inhibitor receptor, signal regulatory protein a (SIRPa), on themselves (Larsson et al., 2016). Blocking CD47 accelerates hematoma removal by promoting erythrophagocytosis by macrophages, and injection of CD47 knockout blood also favors clot resolution when compared to WT blood in ICH (Ni et al., 2016; Jing et al., 2019). In addition to CD47, another scavenger protein, CD36, a well-recognized integral microglia/macrophage cell membrane protein, is involved in the activation of phagocyte-mediated innate immune responses in ICH (Xia et al., 2022). The increase in CD36 expression in the perihematomal region is associated with faster hematoma clearance, and reduced CD36 expression increases the production of pro-inflammatory M1 macrophages/microglia mediators, such as TNF-α and IL-1β, thus inhibiting erythrophagocytosis and hematoma clearance (Fang et al., 2014; Derry et al., 2020). As efferocytosis of eryptotic erythrocytes in HS is also a method of erythrophagocytosis of “abnormal” erythrocytes, the recognition of eryptotic erythrocytes by phagocytes is dependent on Ca^2+^-induced PS exposure on the erythrocyte outer membrane. Similar to apoptotic cells, eryptotic erythrocytes are recognized and subsequently engulfed by PS receptors on macrophages and, thus, rapidly eliminated (Lang et al., 2010).

A recent study has highlighted that, with brain-resident microglia and monocyte-derived macrophages (MDMs), together phagocytose erythrocytes within the hematoma after ICH, MDMs may exhibit a higher phagocytic capacity than others (Chang et al., 2021). Previous studies demonstrated that phagocytosis of apoptotic cells can directly activate anti-inflammatory transcriptional programs in MDMs after acute injury (Hochreiter-Hufford and Ravichandran, 2013). A dramatic shift in MDM gene expression occurs after ICH, and *Axl* is a significantly differentially expressed gene that may primarily contribute to MDMs phenotype transformation (Chang et al., 2018). In addition, the tyrosine kinase family member MERTK was highly expressed in MDMs during both the acute and sub-acute phases after ICH (Chang et al., 2018). The tyrosine kinases AXL and MERTK are PS receptors on MDMs, which are activated by the binding of PS-exposed cells *via* the adaptor proteins, GAS6, and protein S, and promote efferocytosis and trigger anti-inflammatory responses in macrophages (Rothlin et al., 2015). In this study, large amounts of eryptotic erythrocytes in the perihematomal region were the major PS-exposed cells that specifically facilitated reparative MDM responses by AXL/MERTK–mediated efferocytosis, which revealed the novel significance of eryptosis (Chang et al., 2018).

Upon injury, erythrocytes may undergo suicidal death (eryptosis), which is characterized by PS exposure, cell shrinkage, and cell membrane blebbing (Lang and Lang, 2015). Thus, it can be concluded that eryptosis serves a similar significance to apoptosis, that is, the removal of defective, infected, or, otherwise, potentially harmful cells (Elmore, 2007). However, unlike the well-studied apoptosis, knowledge about eryptosis is relatively limited, and there is still much to be learned about the cellular mechanisms involved in eryptosis and its role in the disease. In this review, we summarized the potential correlation between HS and eryptosis, from risk factors to shared molecular mechanisms. At present, few studies validated the occurrence of eryptosis in HS, and its distinct role in the pathogenesis of HS remains uncertain. Further studies are needed to determine the exact extent of erythrocytes, within hematoma/subarachnoid clots, entering eryptosis, and their contribution to the clearance of “abnormal erythrocytes.” Furthermore, how eryptotic erythrocytes interact with inflammatory cells (especially macrophages and microglia) and whether there is a way of mediating eryptosis to benefit neurofunctional recovery after HS warrants further study.

## Author Contributions

MF and FX: conceptualization. YS and CT: validation. YC: investigation. MF: writing—original draft preparation. FX: writing—review and editing. MF and YC: visualization. LM and CY: supervision. XH: project administration. All authors approved the final version of the manuscript.

## Conflict of Interest

The authors declare that the research was conducted in the absence of any commercial or financial relationships that could be construed as a potential conflict of interest.

## Publisher’s Note

All claims expressed in this article are solely those of the authors and do not necessarily represent those of their affiliated organizations, or those of the publisher, the editors and the reviewers. Any product that may be evaluated in this article, or claim that may be made by its manufacturer, is not guaranteed or endorsed by the publisher.
